# Prevalence and molecular epidemiology of *mcr*-mediated colistin-resistance *Escherichia coli* from healthy poultry in France after national plan to reduce exposure to colistin in farm

**DOI:** 10.3389/fmicb.2023.1254122

**Published:** 2023-10-06

**Authors:** Agnès Perrin-Guyomard, Paméla Houée, Pierrick Lucas, Arnaud Felten, Laetitia Le Devendec, Claire Chauvin, Isabelle Kempf

**Affiliations:** ^1^Laboratoire de Fougères, ANSES, Fougères, France; ^2^Laboratoire de Ploufragan-Plouzané-Niort, ANSES, Ploufragan, France

**Keywords:** *mcr*, *Escherichia coli*, poultry, epidemiology, colistin sales

## Abstract

**Introduction:**

Within the 2007–2014 programme for the surveillance of antimicrobial resistance (AMR) in livestock in France, *mcr-1* prevalence average in commensal *Escherichia coli* was found to be 5.9% in turkeys and 1.8% in broilers, indicating that mobile colistin resistance had spread in farm animals. In 2017, the French national Ecoantibio2 plan was established to tackle AMR in veterinary medicine, with the objective of a 50% reduction in exposure to colistin in farm animals within 5 years (from 2014–2015 to 2020). Our objective was to update data concerning the prevalence and molecular epidemiology of colistin resistance, in consideration of colistin sales in poultry production in France.

**Methods:**

Antimicrobial susceptibility of commensal *E. coli* isolated from broilers and turkeys at slaughterhouse was determined by broth micro-dilution. The *mcr* genes were screened by polymerase chain reaction (PCR). Whole genome sequencing (WGS) was used to investigate the genetic diversity of colistin-resistant isolates. Transformation experiments enabled identification of the *mcr*-bearing plasmid replicon types. The correlation between prevalence of colistin resistance and colistin usage data was explored statistically.

**Results and discussion:**

In 2020, in France, the resistance prevalence to colistin in poultry production was 3% in turkeys and 1% in broilers, showing a significant highly positive correlation with a −68% decrease of poultry exposure to colistin since 2014. Only the *mcr-1* gene was detected among the colistin-resistant *E. coli*. More than 80% of isolates are multi-drug resistant with 40% of isolates originating from turkeys and 44% originating from broilers co-resistant to the critically important antimicrobial ciprofloxacin. Most of the strains had no clonal relationship. The *mcr* gene was located in different plasmid types, carrying various other AMR genes. The decrease in colistin resistance among poultry in France can be considered a positive outcome of the national action plans for reduced colistin usage.

## Introduction

Colistin is an antibiotic that has been used for decades in veterinary medicine to control gastrointestinal infections in animals, but also to treat systemic, respiratory or urogenital infections in poultry (Kempf et al., 2013). In France, poultry displayed the greatest exposure to polypeptides, including mainly colistin, measured in ALEA [Animal Level of Exposure to Antimicrobials (Anses, 2022b)], a ratio between the live weight treated and the weight produced (poultry polypeptides ALEA = 0.117), followed by pigs (0.058) and cattle (0.011). In fact, in 2020, colistin consumption was mainly attributed to poultry (45.7%), pigs (30.9%) and cattle (18.4%) (Anses, 2022b). Moreover, polypeptides were the most common class of antibiotics used in broilers and turkeys (representing 30% of the total exposure in ALEA), before mainly penicillins (25%), tetracyclines (20%), sulfonamides (10%) and trimethoprim (8%). After oral administration, the antibiotic is poorly absorbed through the gastrointestinal tract (Mead et al., 2021). Therefore, the intestinal microbiota is subject to selective pressure that can lead to the emergence of colistin resistance. Until 2015, the only known colistin resistance mechanisms were linked to chromosomal mutations affecting the lipopolysaccharide charge, reducing colistin interaction with the outer cell membrane of Gram-negative bacteria. Subsequently, the first mobile colistin resistance gene *mcr-1* was discovered in *Escherichia coli* isolates of various origins, first in China and then worldwide across multiple bacterial species (Liu et al., 2016; Xavier et al., 2016). Since 2015, nine new *mcr* genes (*mcr-2* to *mcr-10*) and subvariants have been described in Enterobacterales (Wang et al., 2020). In the commensal *E. coli* strain collection isolated from livestock in France between 2007 to 2014, *mcr-1* prevalence average was 5.9% in turkeys and 1.8% in broilers, indicating that mobile colistin resistance was already widespread in farm animals (Perrin-Guyomard et al., 2016). Given the dissemination capabilities of *mcr-1*, the high usage levels of colistin in animals and the high percentage of multi-drug-resistant (MDR) isolates in strains harbouring *mcr-1* (Perrin-Guyomard et al., 2016; Mead et al., 2022), the French national Ecoantibio2 (Ecoantibio2, 2017) plan to tackle the risk of antibiotic resistance in veterinary medicine, set up from 2017, had the objective of a 50% reduction in exposure to colistin in food producing animals within 5 years, from 2014–2015 to 2020. Therefore, there was a need to update prevalence and epidemiology data for colistin resistance in poultry production in the country after the implementation of national policies and new scientific findings. For this purpose, *mcr* genes and genetic diversity of strains have been investigated in colistin-resistant *E. coli* originating from broilers and turkeys at slaughterhouses from 2011 to 2020. The correlation between *mcr*-positive isolate prevalence and colistin usage data was explored to evaluate the efficacy of the stewardship programme in poultry production.

## Methods

### Strain collection and antimicrobial susceptibility testing

The sampling framework and collection of *E. coli* isolates were established according to Directive 2003/99/EC and Decision 2013/652/EU on the harmonised monitoring of antimicrobial resistance in zoonotic and commensal bacteria in Europe (European Parliament, 2003; European Commission, 2013). Briefly, every year from 2011 to 2014 (broilers only), then every 2 years from 2014 to 2020 (broilers and turkeys), caecal samples from poultry were collected at slaughterhouses from randomly selected epidemiological units according to a regional stratification plan representing at least 60% of production in France. Caecal content samples were streaked on a chromogenic medium to allow the presumptive isolation of *E. coli* (Perrin-Guyomard et al., 2023). One confirmed *E. coli* from each sample was tested for phenotypic antimicrobial susceptibility by broth micro-dilution using commercial microplates dosed with various concentrations of antimicrobial agents (EUVSEC plates, Thermo Scientific^™^ Sensititre^™^, Dardilly, France). Isolates with a colistin minimum inhibitory concentration (MIC) over the epidemiological cut-off value (Ecoff) of the European legislation (2 mg/L) were selected for further genetic characterisation. MDR isolates, i.e., isolates resistant to at least three of the antimicrobial classes, were enumerated. Resistance to cefotaxime and ceftazidime as well as resistance to ciprofloxacin and nalidixic acid, respectively are addressed together.

### *mcr* screening

Polymerase chain reaction (PCR) assays targeting the *mcr-1* to *mcr-5* genes (Rebelo et al., 2018) and *mcr-6* to *mcr-9* genes (Borowiak et al., 2020) were applied to detect mobile colistin-resistance genes.

### Phylogenetic analyses

*mcr*-positive *E. coli* strains were assigned to one of the main phylo-groups according to the *in silico* approach of the ClermontTyper web tool^1^ (Beghain et al., 2018).

### Transformation

Plasmids from 22 *mcr*-positive isolates were submitted to transformation experiments using electrocompetent *E. coli* DH10B cells (Thermo Scientific, Dardilly, France) and an Eporator Eppendorf device (Dutscher, Brumath, France) according to the manufacturer’s instructions. Firstly, plasmid DNA was extracted from a 1 day Luria-Bertani broth culture (homemade medium) supplemented with colistin sulphate (1 mg/L) (Merck, Saint-Quentin-Fallavier, France) using a NucleoBond Xtra Midi kit (Macherey-Nagel, Hoerdt, France), according to the manufacturer’s protocol. After electroporation, the potential transformants were selected on Mueller Hinton agar (Bio-Rad, Marnes-La-Coquette, France) containing colistin sulphate (2 mg/L). Antimicrobial susceptibility testing and detection of the *mcr* gene were performed on transformants by broth micro-dilution and PCR as described above, respectively.

### PCR-based replicon typing

Total DNA of transformants extracted by boiling were subjected to a PCR-based replicon typing (PBRT) kit (Diatheva, Cartoceto, Italy), according to the manufacturer’s instructions. Amplification products were resolved on 2.5% agarose gel and stained with GelRed Nucleic Acid Gel Stain (VWR, Fontenay-sous-Bois, France).

### Whole genome sequencing

Genomic DNA was extracted from all *mcr*-positive isolates using a QIAGEN DNeasy Blood and Tissue kit (QIAGEN, Courtaboeuf, France). Libraries were prepared for Illumina pair-end sequencing using a Nextera XT DNA Library Preparation kit. Sequencing was performed using a NovaSeq 6000 Illumina platform (Illumina San Diego, CA, USA). Raw sequence data were submitted to the European Nucleotide Archive under study accession no. PRJEB59826.

### Bioinformatics analyses

The reads were assembled with the in-house workflow ARTwork2. Short paired-end reads were trimmed by fastp v0.20.1 (i.e., adapter sequences, mean quality phred score of 25 and correction in overlapped region). Trimmed reads were mapped against the closely related reference genome identified by estimating the Jaccard index with Mash v2.0, among a collection of high-quality fully closed assemblies from Refseq, representative of the species. The samples presenting a depth of coverage against the reference between 50X and 100X were retained, and those higher than 100X were normalised at 100X with Bbnorm v38.22.^2^ Reads were assembled using Shovill v1.1.0.^3^ Contigs smaller than 200 bp or contigs with a coverage less than 2X were removed from the assembly. The closest reference genome was used to perform reference-based scaffolding with MeDuSa v1.6 and gap filling with GMcloser v1.6.2. Then, the finalised assembly was annotated with Bakta v1.5.0. Sequence type (ST) and serotypes were determined using the MLST script^4^ and SeroTypeFinder,^5^ respectively. To detect antimicrobial resistance, the genome was scanned against the ResFinder, PointFinder, and PlasmidFinder databases using staramr v0.7.2. Virulence gene screening was performed using ABRicate v1.01^6^ by querying the VFDB database and VirulenceFinder.^7^ A phylogenetic tree of 48 genome assemblies was built from the number of shared k-mers (kmer size of 17 and sketch size of 10,000) with the in-house script QuickPhylo v1.0.^8^ This tool used Mash v2.0 to compute a distance matrix based on the Jaccard index and cluster sequences with the neighbour-joining (NJ) method. The midpoint approach was used to root the tree.

### Correlation between resistance data and sales

Data on sales and exposure to polymyxins were sourced from annual monitoring of veterinary drugs containing antibiotics in France from 2011 to 2020 (Anses, 2022b). Exposure was measured as a ratio between poultry biomass in kg that could receive polymyxin treatment and poultry biomass produced in the same year (i.e., ALEA). Since these annual figures are available for poultry production, resistance in poultry production was estimated by weighting turkey and broilers resistance data since 2014 by their respective annual biomass (Anses, 2022b).

### Statistical analyses

Trends in colistin resistance over time were evaluated using a Cochran-Armitage test. Spearman rank correlation was calculated between colistin exposure and resistance prevalence in poultry production between 2014 and 2020. The statistical analysis was performed using R 3.6.3 and a two-sided *p*-value 0.05 was considered statistically significant.

## Results

### Trends in colistin resistance and correlation with colistin usage in France

From 2014 to 2020, colistin exposure decrease by 68% in poultry production in France, exceeding the initial −50% objective. In 2020, the ALEA in poultry production was 0.113.

**Figure 1:**
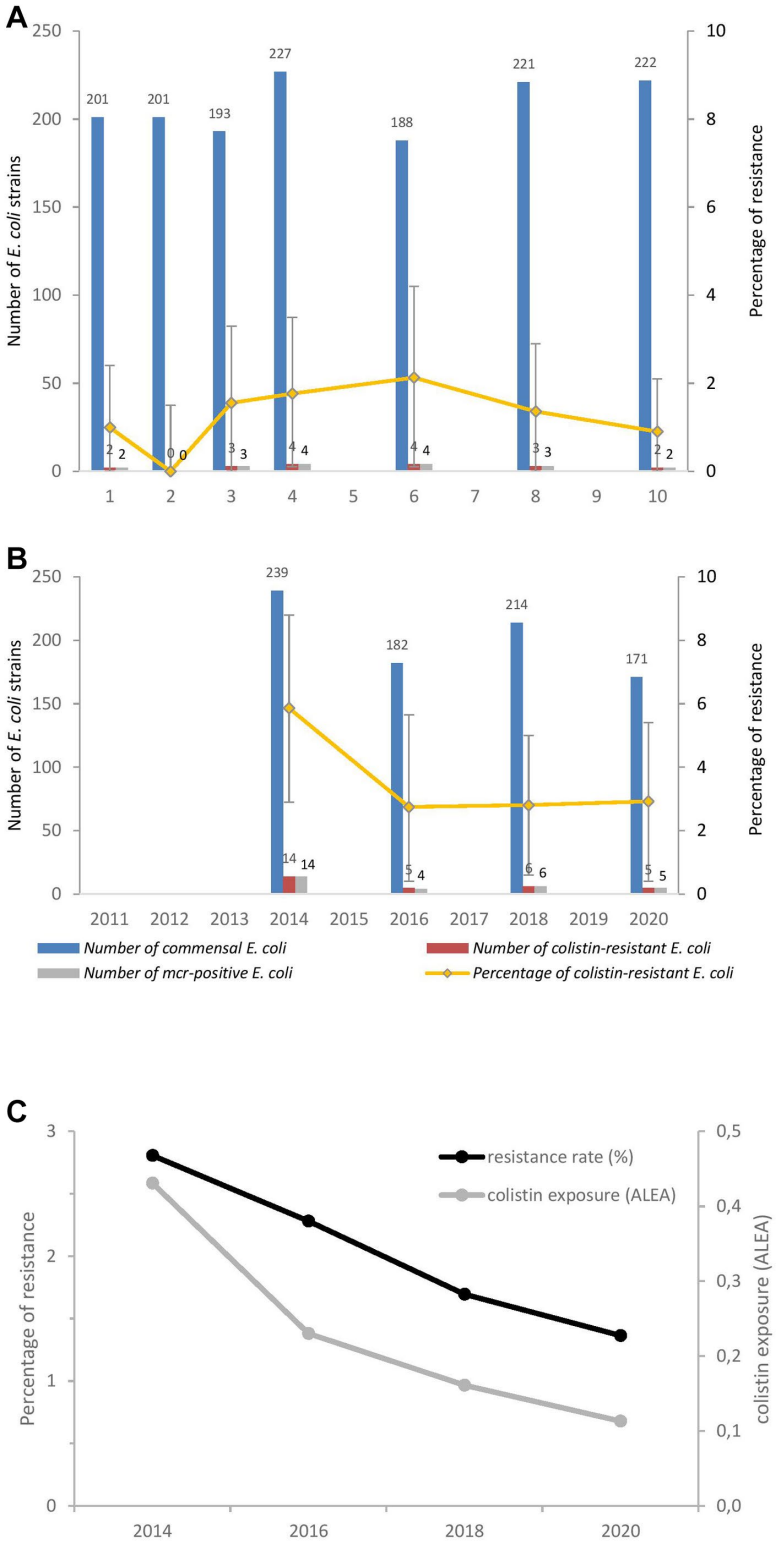
Changes in the proportion of colistin-resistant and *mcr*-positive *E. coli* among commensal *E. coli* isolated from caecal content of broilers **(A)**, turkeys **(B)** and joint evolution of poultry exposure and resistance **(C)**.

Among the commensal *E. coli* isolated from healthy broilers and turkeys between 2011 and 2020, 1.2% (18/1,453) and 3.7% (30/806), respectively were resistant to colistin according to the broth micro-dilution method. The colistin resistance prevalence increased slightly in broilers from 2011 (1%) to 2016 (3%) to reach 1% in 2020 (Figure 1). In turkeys, where the percentage of resistance is higher, prevalence was halved from 2014 (6%) to 2020 (3%) (*p* > 0.05). The resistance prevalence in poultry from 2014 to 2020 was significantly positively correlated with poultry exposure (*r*_s_ = 1) (Figure 1).

### Phenotypic antimicrobial resistance profile of colistin-resistant *Escherichia coli* and *mcr* screening

Among the 48 colistin-resistant isolates, all but one were detected positive for the *mcr-1* gene by PCR. Among the 47 *mcr-1*-positive *E. coli* isolates, 83% from turkeys and 100% from broilers are MDR (Table 1). The most commonly found phenotypic antibiotic resistance pattern according to the Ecoff threshold corresponds to colistin, ampicillin, ciprofloxacin/nalidixic acid, sulfamethoxazole, tetracycline and trimethoprim (16.7%) in turkey isolates and colistin, ampicillin, sulfamethoxazole and trimethoprim in broiler isolates (16.7%). Two strains from turkeys and one from broilers carried up to 8 antibiotic resistances including colistin resistance. In all, 40% of isolates originating from turkeys and 44% originating from broilers exhibited co-resistance to the critically important antimicrobial (CIA) ciprofloxacin. Only one strain was resistant to cephalosporins.

**Table 1:** Phenotypic and genotypic characteristics of colistin resistant *E. coli* isolated from caecal content of broilers and turkeys between 2011 and 2020.

A – Turkeys							
Isolation year	Strain number	Colistin MIC (mg/L)	Phenotypic antimicrobial resistance pattern^a^	Genotypic antimicrobial resistance pattern	Phylogroup	ST	Serotype
2014	14F000712	16	AMP-CAZ-CTX-CIP-COL-NAL-TMP	*bla*_TEM-1B_, *bla*_CMY-2_, *gyrA* (S83L), *mcr-1.1*, *dfrA1*	B2	131	O25:H4
	14F000803	4	AMP-CIP-COL-NAL-SMX-TET-TMP	*bla*_TEM-1B_, *gyrA* (S83L), *mcr-1.1*, *sul2*, tet(A), *dfrA14*	A	48	O8:H11
	14F000811	4	AMP-CHL-CIP-COL-NAL-SMX-TET-TMP	*bla*_TEM-1B_, *cmlA1*, *gyrA* (D87G), *mcr-1.27*, *sul2*, *sul3*, *tet*(B), *tet*(M), *dfrA14*	A	10	O8/O40:H32
	14F000818	8	AMP-CIP-COL-SMX-TET-TMP	*bla*_TEM-1B_, qnrS1, *parE* (I529L) *mcr-1.1*, *sul2*, *tet*(A), *dfrA14*	B2	131	O25:H4
	14F000827	8	AMP-CIP-COL-NAL-SMX-TET-TMP	*bla*_TEM-1B_, *gyrA* (S83L), *mcr-1.1*, *sul1*, *sul2*, *tet*(A), *dfrA1*	D	38	O99:H15
	14F000945	4	AMP-CIP-COL-NAL-SMX-TET-TMP	*bla*_TEM-1B_, *gyrA* (S83L), *mcr-1.1*, *sul1*, *tet*(A), *tet*(B), *dfrA1*	A	10	-:H48
	14F000950	8	AMP-COL-SMX-TET-TMP	*bla*_TEM-1B_, *mcr-1.1*, *sul1*, *sul2*, *tet*(A), *dfrA1*	A	853	O101:H37
	14F000955	8	AMP-CHL-CIP-COL-GEN-NAL-SMX-TET-TMP	*bla*_TEM-1A_, *catA1*, *cmlA1*, *gyrA* (S83L, D87N), *parC* (S80I), *mcr-1.1*, *aac*(3)-IIa, *sul1*, *sul2*, *sul3*, *tet*(A), *tet*(B), *dfrA1*	B1	533	O132:H10
	14F000956	8	AMP-COL-TET-TMP	*bla*_TEM-1B_, *mcr-1.1*, *tet*(A)	G	117	O161:H4
	14F001009	8	COL-TET	*mcr-1.1*, *tet*(B)	G	117	O24:H4
	14F001453	8	COL-TET	*mcr-1.1*, *tet*(B)	A	48	O5:H10
	15F000312	8	AMP-COL-TET	*bla*_TEM-1C_, *parE* (I529L), *mcr-1.1*, *tet*(A)	B2	131	O25:H4
	15F000314	8	AMP-CIP-COL-NAL-SMX-TET	*bla*_TEM-1C_, *gyrA* (S83L, D87N), *parC* (S80I, E84G), *parE* (I355T), *mcr-1.1*, *sul3*, *tet*(A)	F	354	O153:H34
	15F000337	8	AMP-CIP-COL-NAL-SMX-TET-TMP	*bla*_TEM-1B_, *gyrA* (S83L, D87N), *parC* (S80I), *parE* (S458A), *mcr-1.1*, sul1, *sul2*, *tet*(A), *dfrA1*	A	617	O101:H9
2016	16F000703	4	AMP-COL-SMX-TET	*bla*_TEM-1B_, *mcr-1.1*, *sul2*, *tet*(B)	D	108	O13/O129:H15
	16F000734	4	AMP-AZM-CHL-CIP-COL-NAL-SMX-TET-TMP	*bla*_TEM-1B_, *mph*(A), *cmlA1*, *gyrA* (S83L, D87N), *parC* (S80I), *mcr-1.1*, *sul1*, *sul2*, *sul3*, *tet*(A), *tet*(B), *dfrA1*, *dfrA17*	B1	2599	O21:H2
	16F000778	8	AMP-CHL-COL-SMX-TET	*bla*_TEM-1B_, *cmlA1*, *mcr-1.1*, *sul3*, *tet*(A)	A	10	O9a/O9:H9
	16F001585	4	AMP-COL-SMX-TET-TMP	*bla*_TEM-1B_, *mcr-1.1*, *sul2*, *tet*(A), *dfrA14*	A	1632	O182:H38
	17F000155	4	AMP-COL-SMX-TET-TMP	*bla*_TEM-1B_, *sul1*, *tet*(A), *dfrA1*	B2	12	O4:H4
2018	18F000932	8	AMP-CHL-COL-SMX-TET-TMP	*bla*_TEM-1B_, *cmlA1*, *mcr-1.1*, *sul1*, *sul2*, *sul3*, *tet*(A), *dfrA1*	A	43	O6:H10
	18F000939	4	AMP-COL-SMX-TET-TMP	*bla*_TEM-1B_, *mcr-1.1*, *sul2*, *tet*(A), *dfrA1*	D	5135	O167:H26
	19F000038	8	AMP-CIP-COL-TET	*bla*_TEM-1B_, qnrS1, *mcr-1.1*, *tet*(A)	D	349	O166:H15
	19F000042	8	AMP-COL	*bla*_TEM-1B_, *mcr-1.1*	A	1137	O88:H38
	19F000113	8	AMP-CHL-COL-TET-TMP	*bla*_TEM-1B_, *cmlA1*, *mcr-1.1*, *tet*(A), *dfrA12*	A	1716	O130:H26
	18F000491	8	AMP-COL-TET	*bla*_TEM-1B_, *mcr-1.1*, *tet*(A)	D	69	O21:H6
2020	20F000574	4	AMP-COL-GEN-SMX-TET-TMP	*bla*_TEM-1A_, *mcr-1.1*, *aac*(3)-IIa, *sul1*, *sul2*, *tet*(A), *dfrA1*	B1	3580	O109:H7
	20F000951	4	AMP-COL	*bla*_TEM-1B_, *mcr-1.1*	A	1303	O130:H18
	20F000954	8	AMP-CIP-COL-NAL-TET	*bla*_TEM-1B_, *gyrA* (D87G), *mcr-1.1*, *tet*(A)	D	686	O26:H34
	21F000145	4	AMP-COL	*bla*_TEM-1B_, *mcr-1.1*	D	5451	-:H19
	21F000146	8	AMP-CHL-COL-SMX-TET	*bla*_TEM-1B_, *catA1*, *cmlA1*, *mcr-1.1*, *sul3*, *tet*(A)	A	–	O132:H32

B – Broilers							
Isolation year	Strain number	Colistin MIC (mg/L)	Phenotypic antimicrobial resistance pattern^a^	Genotypic antimicrobial resistance pattern	Phylogroup	ST	Serotype
2011	12F000020	8	AMP-COL-SMX-TET-TMP	*bla*_TEM-1B_, *mcr-1.1*, *sul2*, *tet*(A), *dfrA1*	A	10	O129/O13:H4
	12F002303	8	AMP-CHL-COL-SMX-TET-TMP	*bla*_TEM-1B_, catA1, *cmlA1*, *mcr-1.1*, *sul1*, *sul3*, *tet*(A), *tet*(M), *dfrA1*	B1	212	O18ac/O18:H49
2013	13F001905	4	AMP-CIP-COL-NAL-SMX-TET-TMP	*bla*_TEM-1B_, *gyrA* (S83L), *mcr-1.1*, *sul1*, *sul2*, tet(A), *dfrA1*	A	10	O13/O129:H48
	13F002282	8	AMP-COL-TMP	*bla*_TEM-1B_, *mcr-1.1*	A	1286	O117:H42
	14F000177	8	AZI-CHL-CIP-COL-NAL-TET-TMP	*gyrA* (S83L), *parC* (S80I), *mcr-1.1*, *tet*(M), *dfrA12*	A	10	O127:H21
2014	14F000716	4	CHL-COL-SMX-TET-TMP	*catA1*, *cmlA1*, *mcr-1.1*, *sul1*, *sul3*, *tet*(A), *dfrA1*	B1	13418	O23:H16
	14F000967	8	AMP-COL-TET	*bla*_TEM-1B_, *mcr-1.1*, *tet*(A)	F	1485	O83:H42
	14F000968	4	AMP-COL-TET	*bla*_TEM-1B_, *mcr-1.1*, *tet*(A)	A	48	O88:H16
	15F000320	8	AMP-CIP-COL-NAL-TET	*bla*_TEM-1B_, *gyrA* (S83L, D87N), *parC* (S80I), *parE* (S458A), *mcr-1.1*, *tet*(B)	B1	1196	O91:H28
2016	16F000741	4	AMP-COL-NAL-SMX-TET-TMP	*bla*_TEM-1B_, *mcr-1.1*, *gyrA* (D87G), *sul1*, *sul2*, *tet*(A), *dfrA1*	A	10	O145:H40
	17F000026	4	AMP-CHL-CIP-COL-NAL-SMX-TET-TMP	*bla*_TEM-1B_, catA1, *gyrA* (S83A), *mcr-1.1*, *sul1*, *tet*(A), *dfrA1*	C	23	O76/O8:H9
	17F000069	4	CIP-COL-NAL-TET	*gyrA* (S83L, D87N), *parC* (S80I)*mcr-1.1*, *tet*(A)	B1	1800	O82:H21
	16F001469	4	AMP-COL-SMX-TMP	*bla*_TEM-1B_, *mcr-1.1*, *sul2*, *dfrA1*	A	1137	O85:H48
2018	18F001603	8	CIP-COL-NAL-TET	*gyrA* (S83L), *parC* (S80I), *mcr-1.1*, *tet*(A)	F	1485	O83:H42
	18F000257	4	AMP-AZI-CHL-CIP-COL-NAL-SMX-TET-TMP	*bla*_TEM-1B_, mph(A), catA1, *gyrA* (S83L, D87N), *parC* (A56T, S80I), *mcr-1.1*, *sul1*, *sul2*, *tet*(A), *tet*(B), *dfrA14*, *dfrA17*	A	744	O101:H10
	18F000469	4	AMP-CIP-COL-NAL-SMX-TET	*bla*_TEM-1B_, *gyrA* (S83L), *parC* (S80I), *mcr-1.1*, *sul2*, *tet*(A)	F	1485	O83:H42
2020	20F000375	8	AMP-COL-SMX-TMP	*bla*_TEM-1B_, *mcr-1.1*, *sul1*, *sul2*, *dfrA1*	A	2223	O75:H42
	20F000380	8	AMP-COL-SMX-TMP	*bla*_TEM-1B_, *mcr-1.1*, *sul2*, *dfrA1*	A	10	-:H48

^a^Ampicillin, AMP; Azithromycin, AZM; Cefotaxime, CTX; Chloramphenicol, CHL; Ciprofloxacin, CIP; Colistin, COL; Gentamicin, GEN; Nalidixic Acid, NAL; Sulfamethoxazole, SMX; Tetracycline, TET; Trimethoprim, TMP.

### Genotypic antimicrobial resistance profile of colistin-resistant *Escherichia coli*

Whole genome sequencing analyses confirmed the presence of the *mcr-1* gene in 47 isolates (*mcr-1.1*, *n* = 46; *mcr-1.27*, *n* = 1), and the absence of any known *mcr* gene in the turkey 17F000155 isolate. No mutations in the two component systems PmrAB and PhoPQ inducing chromosomal colistin resistance could be detected in this strain, nor in the other 47 strains. Colistin MIC value for 17F000155 isolate is within the one two-fold dilution step from the Ecoff that could explain why no resistance gene was found. Concerning the other antibiotic classes, phenotypic results correlated with the resistance determinants characterised by WGS, except for one broiler strain resistant to azithromycin and chloramphenicol and two strains, in each poultry species, resistant to trimethoprim by broth micro-dilution (Table 1). MIC value for these antimicrobials are within the one two-fold dilution step from the Ecoff that could explain why no resistance gene was found.Mutations in the quinolone resistance-determining region of *gyrA* (S83L, S83A, D87N or D87G) associated or not with mutations in those of *parC* (A56T, E84G or S80I) and *parE* (I529L, I355T or S458A) were detected in ciprofloxacin/nalidixic acid-resistant strains, leading to clinical ciprofloxacin resistance for 13 out of 20 isolates. Among these 13 strains, three originating from turkeys had only one mutation in *gyrA* or *parE*, conferring resistance to nalidixic acid only, if occurring alone. This phenotypic resistance to ciprofloxacin, could be due to an additional decreased expression of outer membrane porins and/or overexpression of multidrug efflux pumps or the presence of the additional plasmid-mediated quinolone resistance *qnrS* gene for one of them. Only one colistin-resistant strain isolated from turkeys in 2014 was co-resistant to cephalosporins, and contained a *bla*_CMY-2_ gene.

### Molecular epidemiology of *mcr*-mediated colistin-resistance *Escherichia coli*

**Figure 2:**
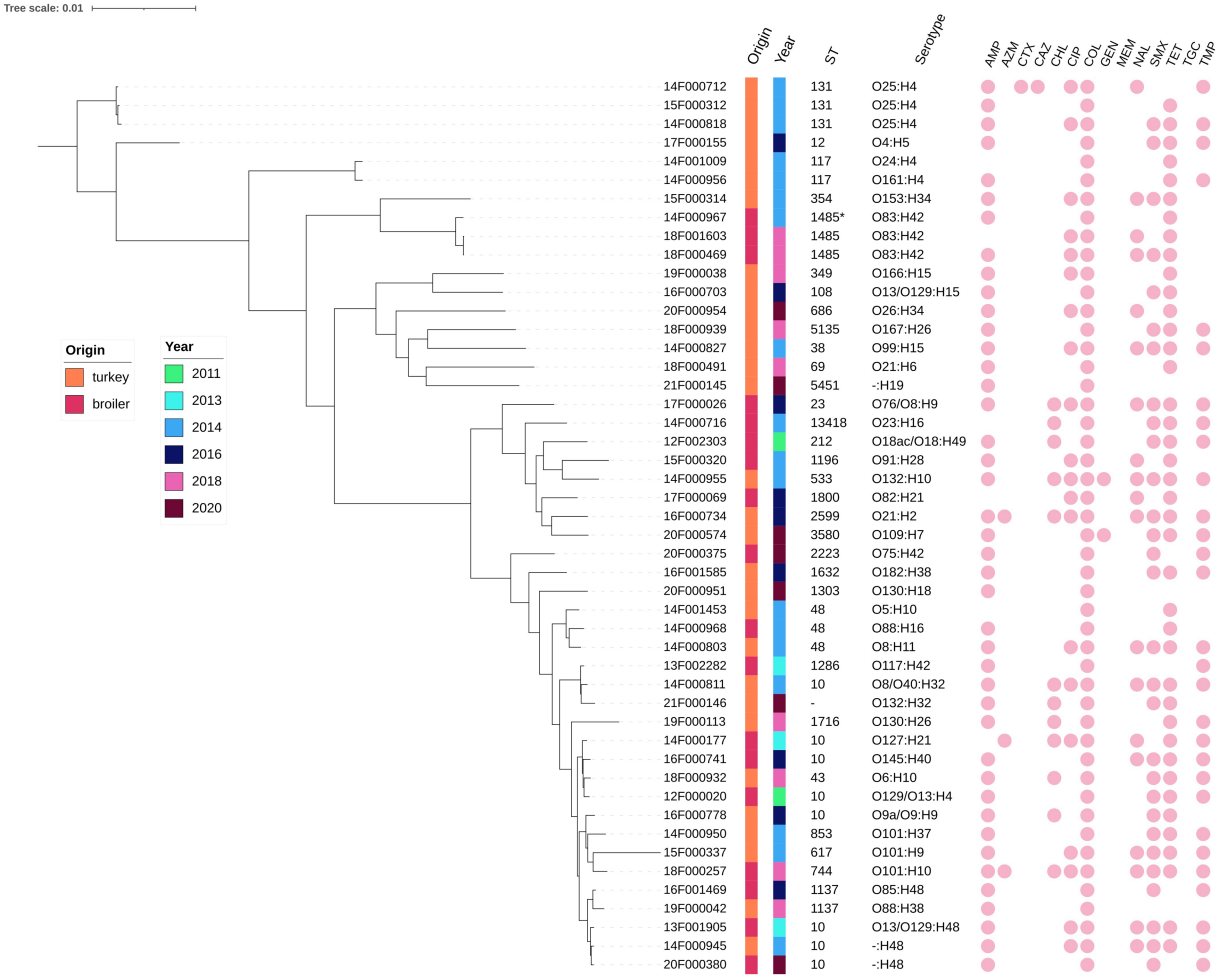
Phylogenetic tree of the *mcr*-positive *E. coli* isolates from caecal content of broilers and turkeys between 2011 and 2020 (WGS).

The isolates belonged to a variety of serotypes (16 for broilers, 28 for turkeys) and multilocus sequence typing (MLST) schemes (12 for broilers, 24 for turkeys) (Figure 2). ST10 was the most frequent sequence type in poultry strains (8/48), followed by ST1485 combined with serotype O83:H42 in broilers (3/18) and ST131 combined with serotype O25:H4 in turkeys (3/30). Concerning phylogroups, the phylogroups from A, B1, B2, D, F and G were represented in the *mcr-1*-positive *E. coli* from turkeys, whereas in broilers, only groups A, B1, C and F were detected (Table 1). Regardless of the animal origin, most isolates belonged to group A (43.3% and 55.6% respectively), followed by group D in turkeys (23.3%) and group B1 in broilers (22.2%). According to the phylogenetic tree, most strains have no clonal relationship, but a few (e.g., 14F000712/15F000312/14F000818, 18F001603/18F000469, and 13F001905/14F000945/20F000380), belonging to the same sequence type and to the same serotype, are very closely related even though they were isolated from different flocks and poultry species or during different years (Figure 2).

Transformation experiments were performed with 13 isolates from turkeys and 9 isolates from broilers in order to identify the *mcr-1*-bearing plasmid replicon types (Table 2). The colistin MICs of transformants were 2 to 4 times higher than the colistin MICs of the recipient strain (1 mg/L). PCR experiments confirmed the presence of *mcr-1* in transformants. Five different replicon types (IncHIA, IncX4, IncI2, IncI1α and IncFII) were found in *mcr-1* transformants. IncHI2 was predominant among colistin-resistant transformants originating from both species of poultry. IncI2 and IncX4 were the second most frequent plasmid types found in broilers and turkeys, respectively. Two multi-replicon plasmids (HI2/FII and HI2/FII/Iα) were recovered from transformants originating from broiler or turkey *mcr-1*-positive *E. coli* isolates. Transformants with the IncHI2-type plasmid acquired other antimicrobial phenotypic resistances, such as ampicillin, tetracycline, trimethoprim and/or sulfamethoxazole resistances, whereas transformants harbouring X4 and I2-type plasmids were found to be resistant only to colistin (Table 2).

**Table 2:** Antimicrobial resistance pattern of transformants harbouring an *mcr-1* bearing plasmid originating from strains isolated from turkeys (A) and broilers (B).

(A) Turkeys			
Isolation year	Transformant^a^	*mcr-1* bearing plasmid	Antibiotic resistance pattern^b^
2014	14F000712_T	HI2	AMP-COL-TMP
	14F000803_T	I1α	AMP-COL
	14F000818_T	X4	COL
	14F000827_T	X4	COL
	14F000950_T	I1α	AMP-COL
	14F000956_T	FII	AMP
	14F001009_T	X4	COL
	15F000312_T	X4	COL
	15F000314_T	HI2	AMP-COL-SMX-TET
	15F000337_T	HI2	COL-TET
2016	16F000734_T	HI2 + I1α + FII	CHL-COL-SMX-TET-TMP
	16F000778_T	HI2	AMP-COL
	16F001585_T	I2	COL

(B) Broilers			
Isolation year	Transformant^a^	*mcr-1* bearing plasmid	Antibiotic resistance pattern^b^
2011	12F002303_T	HI2	AMP-CHL-COL-SMX-TET-TMP
2013	13F001905_T	HI2	AMP-COL-SMX-TMP
	13F002282_T	I1α	AMP-COL
2014	14F000716_T	HI2 + FII	AMP-COL-SMX-TMP
	14F000968_T	I2	COL
	15F000320_T	I2	COL
2016	16F000741_T	HI2	AMP-COL-SMX-TMP
	16F001469_T	I2	COL
	17F000069_T	I2	COL-TET

^a^The code of transformants is given after the original strain code;^b^Ampicillin, AMP; Chloramphenicol, CHL; Colistin, COL; Sulfamethoxazole, SMX; Tetracycline, TET; Trimethoprim, TMP.

## Discussion

This study aimed to deepen our understanding of the prevalence and genotypic characteristics of colistin-resistant indicator *E. coli* isolated from broilers and turkeys sampled at slaughterhouses in France, in consideration of sales of colistin for the poultry sector. This analysis was made possible as strains were derived from the European monitoring programme of antibiotic resistance in food producing animals conducted each year, with the same representative sampling of poultry production in the country, e.g., sampling design and sample size, and the same analytical methods. Sales data were collected annually from marketing authorisation holders in a standardised and consistent process (Anses, 2022b).

Our phenotypic results indicate that percentages of colistin resistance are rather low in poultry, but three times higher in turkeys than in broilers. This difference in colistin resistance levels between the two poultry species is also reported in other European countries with ratios ranging from 4 in Italy (5.9% in broilers, 22.9% in turkeys) to 9 in Portugal (3% in broilers, 27% in turkeys) and 15 times more in Poland (0.2% in broilers, 3.1% in turkeys) in 2014 (Alba et al., 2018; Clemente et al., 2019; Zając et al., 2019). An attempt to distinguish between turkeys and broilers exposure from sales data, showed that polymyxins exposure was higher in turkeys than in broilers (Anses, 2022b). This observation was confirmed in parallel by on-farm data collected by professionals (Rousset, 2019; Zając et al., 2019) speculated that the difference in colistin resistance between broilers and turkeys could be related to the longer life span of turkeys, and consequently to a longer length of exposure to colistin treatment, favouring antibiotic resistance selection. During the monitoring period, the number of colistin-resistant *E. coli* decreased in turkeys, but this decrease was not significant. These findings are consistent with the general trends reported in Europe among food-producing animals, where levels of colistin resistance have decreased since 2014 in isolates from broilers and turkeys to reach median levels of rare in broilers (0%) to low in turkeys (1.2%) in 2020 (European Food Safety Authority and European Centre for Disease Prevention and Control, 2022). In veterinary medicine in France, similar decreasing trends have also been observed from diseased animals since 2007. In 2020, the proportions of non-susceptible *E. coli* isolates collected from various pathologies reached less than 3% in turkeys and 2% in broilers (Anses, 2022a). The decrease in resistance observed between 2014 and 2020 could be attributed to lower sales volumes observed in the same period. A significant correlation between polymyxin exposure and colistin resistance was observed from our data. A significant relationship has also been reported in Europe from annual surveillance data analysis (European Centre for Disease Prevention and Control (ECDC) European Food Safety Authority (EFSA) and European Medicines Agency (EMA), 2021). In the same way, ban of avoparcin as growth promoter in Denmark in 1995 statistically highly significant declined vancomycin-resistant *Enterococcus faecium* isolated from broilers during 1995–1998 (Bager et al., 1999). Outside Europe, colistin withdrawal policy after 2015 have also had a significant effect on reducing colistin resistance in both animals and humans in China for 4 years (Wang et al., 2020). The decrease in resistance in our study can therefore be considered an outcome of the stewardship measures adopted in France for reduced colistin usage, which included development of resistance screening method for veterinary diagnostic laboratories (Jouy et al., 2017), publication of scientific opinions in addition to official European (European Medicine Agency (EMA) and European Surveillance of Veterinary Antimicrobial Consumption (ESVAC), 2016) and national reduction targets (Ecoantibio2, 2017). These specific measures were also supported by generic ones applying to all antimicrobial classes, in line with the two Ecoantibio national plans (Urban et al., 2023). Both antimicrobial usage monitoring systems providing information in poultry production in France reported a considerable decrease in polymyxin exposure, −70% between 2011 and 2020 based on poultry sales (Anses, 2022b), −79% in broilers and −57% in turkeys between 2018 and 2020, considering on-farm professional data collection (Rousset et al., 2022). The official national objective of −50% in colistin sales between 2014/2015 and 2020 included in Ecoantibio2 plan (Ecoantibio2, 2017) was achieved and surpassed, with a −68% reduction achieved in poultry. In the same period total antimicrobial consumption in poultry production decreased by 55%. These results highlight engagement and voluntary adhesion of all professionals to the reduction objectives in the absence of specific regulation.

All but one colistin-resistant strains harboured the mobile gene *mcr-1*. None of the *mcr-2* to *mcr-9* genes were detected in our study. These findings support the reported data on the prevalence of *mcr* genes from livestock animals. Moreover, recent meta-analyses including publications concerning *mcr*-mediated colistin-resistant *E. coli* have shown that *mcr-1* is the most predominant and abundant variant worldwide, especially in poultry isolates (Bastidas-Caldes et al., 2022; Shi et al., 2023). Nevertheless, not all studies have determined the presence of all the reported variants of the *mcr* genes. This bias in the screening could explain why the other variants of the *mcr* genes have been less frequently reported.

Most colistin-resistant strains are MDR, with various resistance patterns. These phenotypic antimicrobial resistance patterns are explained by antimicrobial resistance genes, except in a few cases, where MICs of colistin, azithromycin, chloramphenicol or trimethoprim were one two-fold dilution step higher than the Ecoff, which could explain why no gene was found by WGS. In addition to the high diversity in antimicrobial resistance patterns, multiple serotypes, phylotypes and ST types were encountered among isolates of both turkey and broiler origin, demonstrating a wide range of *E. coli* strains harbouring *mcr*-plasmids. The distribution of phylotypes encountered in microbiota of animals is usually more diverse than in humans (Smati et al., 2015). High counts of phylogroup A associated with variable proportion of other group are a characteristic of the enterocolitype 3, often host-specific to chicken (Pavlickova et al., 2015; Smati et al., 2015) whereas phylogroup B2 is dominant in commensal human strains in developed countries (Tenaillon et al., 2010). From a one health perspective, it is important to highlight that three *mcr-1-*positive strains isolated from turkeys in 2014, were characterised as ST131 O25:H4, belonging to the phylogenetic group B2. *E. coli* sequence type 131 is a worldwide pandemic clone, causing predominantly community onset antimicrobial-resistant infections in humans (Rogers et al., 2011). This clone is an extra-intestinal pathogenic *E. coli*, initially described amongst *E. coli* harbouring the *bla*_CTX-M-15_ ESBL gene and also amongst fluoroquinolone-resistant non-ESBL-producing isolates. The ST131 strains isolated in our surveillance programme were MDR, with resistance to CIAs such as fluoroquinolones and/or cephalosporins. These isolates also contained various virulence factors, including *traT*, *ompT*, *iutA*, *fimH* and *kpsM II* implicated in bacterial survival, invasiveness or adhesion (Riley, 2014) and are a microbiological hazard for humans through the food chain.

IncI2, IncX4 and IncHI2 accounted for 77% of plasmid types driving the *mcr-1* genotype in our poultry isolates. This is consistent with other reported findings on *mcr-1* plasmid location for strains of poultry origin. From 80 *mcr-1-*positive *E. coli* isolated from food-producing broilers and turkeys in Poland between 2011 and 2016, the *mcr-1* gene was detected on the same contig as IncX4 (76.3%) and IncHI2 (6.3%) replicons (Zając et al., 2019). In another study, the *mcr-1* gene was located on IncX4 and IncHI2 plasmids in *E. coli* from broilers in the Netherlands (Veldman et al., 2016). In the environment of German poultry slaughterhouses, Savin *et al* have demonstrated that *E. coli* carrying *mcr-1* isolated from process waters and wastewater were mostly affiliated with IncX4 (37%), IncI1 (21%) and IncHI2 (21%) plasmids (Savin et al., 2020). IncI plasmids are also described in Europe predominantly in *E. coli* isolated from poultry sources (Rozwandowicz et al., 2018). *In vitro* competition assays have demonstrated that *mcr-1* bearing IncI2, IncX4 and IncHI2 plasmids were quite stable and would not affect the growth of their bacterial hosts (Wu et al., 2018). This could explain why these plasmids were most often found in colistin-resistant *mcr-1*-containing *E. coli* of poultry origin. Moreover, *mcr-1* bearing IncHI2-type plasmids are often associated with other antimicrobial resistance genes, as shown in our study. This means that in spite of the reduction of colistin use in poultry, selective pressure with other antimicrobial treatments like ampicillin may help to maintain plasmid-mediated colistin resistance and multidrug resistance spread. Resistance to CIAs like fluoroquinolones in most *mcr*-positive strains could be a major public health hazard towards therapeutic options. Conversely, *mcr-1* bearing plasmids belonging to the IncX4 and IncI2 rarely carry antimicrobial resistance genes other than *mcr-1*, as demonstrated in our study. Nevertheless, plasmids IncX and IncI are narrow-host range plasmids, mainly isolated from *E. coli* from animal sources.

## Conclusion

In broiler and turkey productions in France, we could detect only the *mcr-1* gene in different lineages of colistin resistant *E. coli* isolated at random and non-selective from caecal content of healthy poultry. This gene is located in different plasmid types, carrying various other antimicrobial resistance genes. Different replicons associated with the *mcr-1* gene were identified, including mainly IncHI2/IncI2/X4. Many isolates were MDR, highlighting the potential of colistin resistance to be co-selected by several other antibiotics. Nevertheless, the drop in colistin sales, as well as those of the other main antibiotics used in veterinary medicine, is helping to reduce the selection pressure for colistin resistance. These results, from an ecological design not establishing causal link, reflect the positive impact of the various action plans for the reduction of antibiotic use in the veterinary field and the importance of antibiotic resistance surveillance programmes.

## Data availability statement

The datasets presented in this study can be found in online repositories. Raw sequence data were submitted to the European Nucleotide Archive under study accession no. PRJEB59826.

## Author contributions

APG: Writing – original draft. PH: Writing – review & editing. PL: Writing – review & editing. AF: Writing – review & editing. LD: Writing – review & editing. CC: Writing – original draft. IK: Writing – original draft.

## Footnotes

^1^ http://clermontyping.iame-research.center

^2^ https://jgi.doe.gov/data-and-tools/bbtools/

^3^ https://github.com/tseemann/shovill

^4^ https://github.com/tseemann/mlst

^5^ https://cge.food.dtu.dk/services/SerotypeFinder/

^6^ https://github.com/tseemann/abricate

^7^ https://cge.food.dtu.dk/services/VirulenceFinder/

^8^ https://github.com/afelten-Anses/QuickPhylo
